# Investigation of the Frequency of Femoroacetabular Impingement Using Computed Tomography Images

**DOI:** 10.7759/cureus.99992

**Published:** 2025-12-24

**Authors:** Kemal Yazici, Muhammed Yasir Altunisik, Burak Aydin, Seyran Kılınç, Zekeriya Öztemur

**Affiliations:** 1 Orthopaedics and Traumatology, Merzifon Kara Mustafa Paşa Devlet Hastanesi, Amasya, TUR; 2 Orthopaedics and Traumatology, Sivas Cumhuriyet University, Sivas, TUR

**Keywords:** cam, femoroacetabular impingement, hip, mix, pincer

## Abstract

Objective: The aim of this study is to investigate retrospectively the prevalence of cam and pincer-type femoroacetabular impingement (FAI) in the adult patient population in Turkey using proven measurement methods on computed tomography (CT) images of the patients.

Methods: This study included 273 patients between the ages of 20 and 51. Previously taken CT images of the patients were retrospectively reviewed. Alpha angle (AA), femoral head neck offset (FHNO), lateral centre-edge angle (LCEA), and acetabular version angle (AVA) measurements were made on these images. In addition, the patients were evaluated for the presence of the crossover sign.

Results: Of the patients, 160 (58.6%) were male, 113 (41.4%) were female, and the mean age of the patients was 36.21±9.05 (20-51) years. While CS was seen in 15% (41 patients) of the patients, it was not seen in 85% (232 patients). As a result of the measurements, the AA average was 55.43°±10.17°, FHNO average was 8.19±1.91 mm, LCEA average was 35.22°±6.59°, and AVA average was 17.71°±5.19°. AA was measured at 55° and above in 46.5% (127 patients), and FHNO was measured below 8 mm in 38.8% (106 patients). LCEA was measured at 40° and above in 21.6% of patients (59 patients), and AVA was measured at less than 15° in 29.7% of patients (81 patients).

Conclusion: In the measurements we made using accepted diagnostic accuracy methods, the prevalence of FAI was found to be cam type at 25.3%, pincer type at 25.3%, and mixed type at 23.8%. Although it varies between societies, FAI is a common morphological change in the general population. FAI syndrome is diagnosed by supporting this change with the clinic. When making this diagnosis, unnecessary treatments can be prevented by considering the prevalence of FAI in societies.

## Introduction

There are many causes of hip pain in the adult patient population, but osteoarthritis is the most common cause. Femoroacetabular impingement (FAI) syndrome is more common, especially in young adults and athletes, and is associated with the development of osteoarthritis [1].

FAI is a chronic condition in which there is abnormal contact between the femoral head and acetabulum across the hip joint due to disorders of the femoral head and acetabulum [2]. FAI was first described by Ganz et al. [3]. This abnormal contact in FAI occurs more frequently during extreme ranges of motion of the hip, especially during flexion and internal rotation movements. After repetitive movements, this femoroacetabular contact creates excessive stress on the labrum and articular cartilage. As a result of this stress, degeneration and tearing of the acetabular labrum, damage to the articular cartilage, and osteoarthritis may occur [3].

FAI is divided into three types according to the anatomical features of the pathology: cam, pincer, and mixed type. This classification is made according to the characteristic morphological changes of the bone structures. Cam morphology occurs due to the decrease in the anterior femoral head neck offset or due to an abnormally shaped femoral head. Pincer type occurs due to excessive coverage of the femoral head, due to abnormalities on the acetabular side. Mixed type is seen in cases where both cam morphology and pincer morphology are present at the same time. Some radiological measurements and markers are used in the diagnosis of FAI. In cam morphology, alpha angle (AA) and femoral head neck offset (FHNO) measurements are used, while in pincer morphology, lateral centre-edge angle (LCEA) and acetabular version angle (AVA) measurements and the presence of the crossover sign (CS) are used [1,2]. In the studies, the values ​​of AA >55°, FHNO <8 mm, LCEA>40°, and AVA <15° were considered significant in terms of FAI [4,5].

The aim of this study is to determine the prevalence of cam- and pincer-type FAI in the adult patient population in Turkey retrospectively using proven measurement methods on computed tomography (CT) images of patients.

## Materials and methods

This study was conducted with the approval of the local ethics committee dated 22.02.2023 and numbered 2023-02/14. The sample size in our study was calculated as 273 out of 5,000 patients with a prevalence of 75% and a deviation of 5% using the prevalence found in a previous systematic review study with OpenEPI-updated [6,7]. The study included 273 patients selected by a systematic sampling method among patients aged 20-51 who underwent lower abdominal and pelvic tomography at Health Services Practice and Research Hospital between 01.01.2020 and 31.12.2022. Patients under 20 and over 51 years of age, those with femoral neck or acetabular fractures, those who had undergone surgery in this area, and those with osteoarthritis findings in their imaging were excluded from the study. Informed consent was obtained from the patients we included in the study.

**Figure 1:**
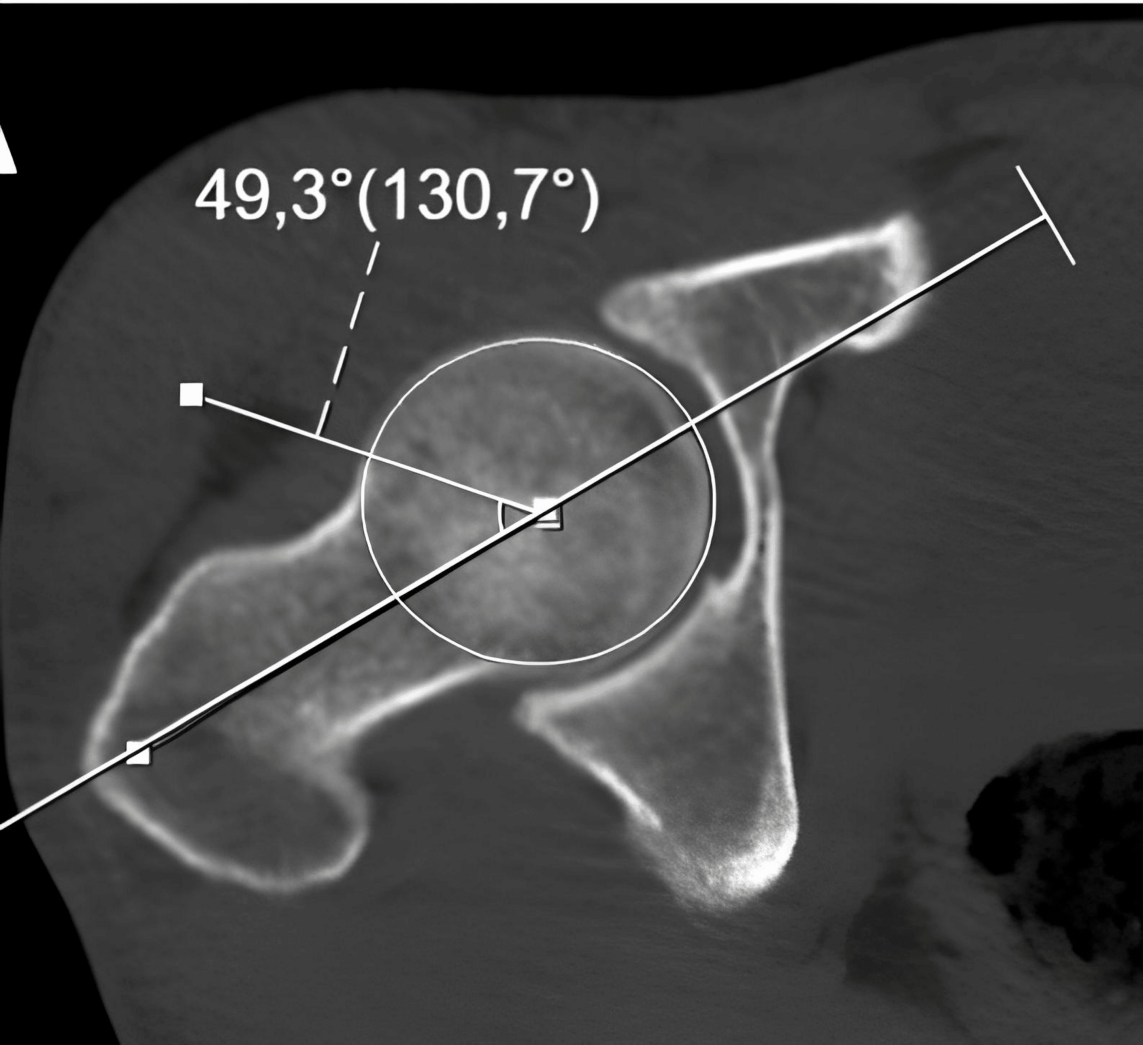
Alpha angle (AA) measurements

**Figure 2:**
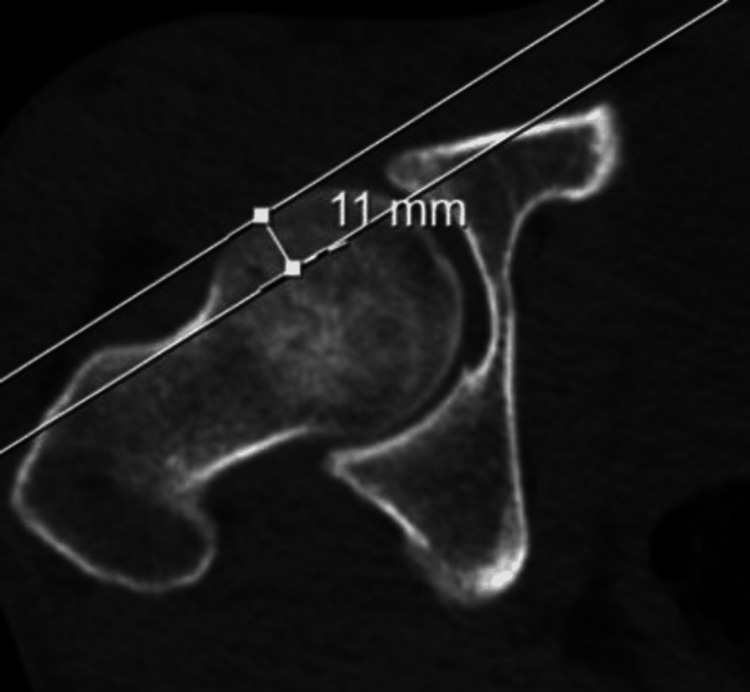
Femoral head neck offset (FHNO) measurements

**Figure 3:**
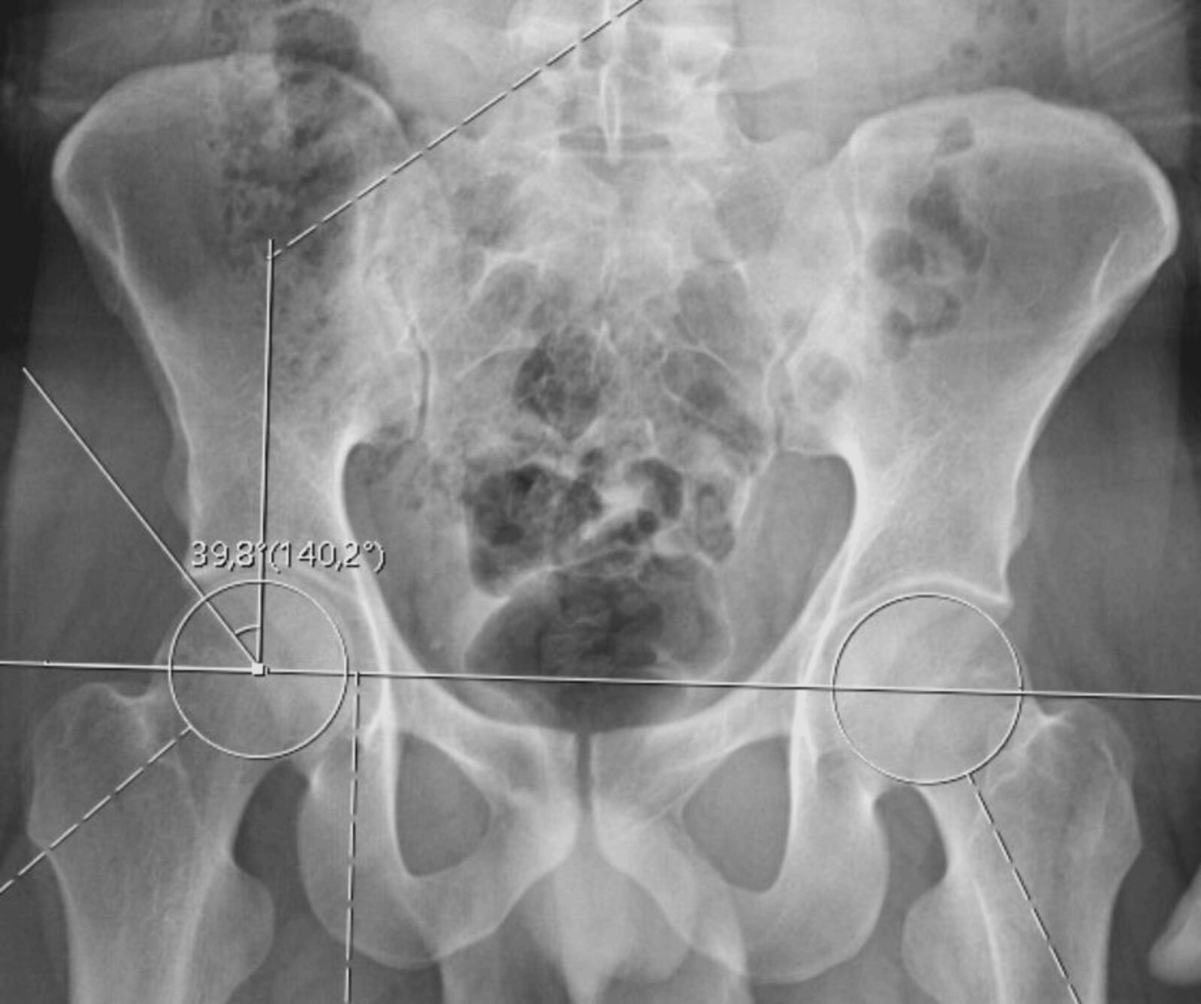
Lateral centre-edge angle (LCEA) measurements

**Figure 4:**
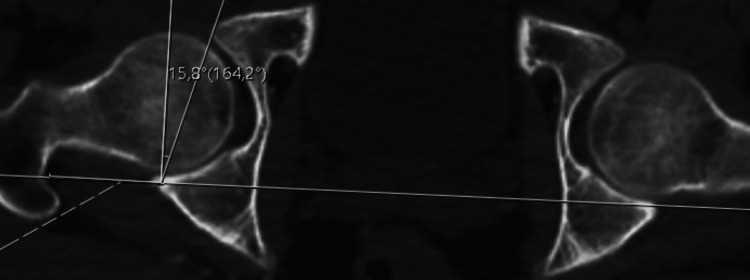
Acetabular version angle (AVA) measurements

**Figure 5:**
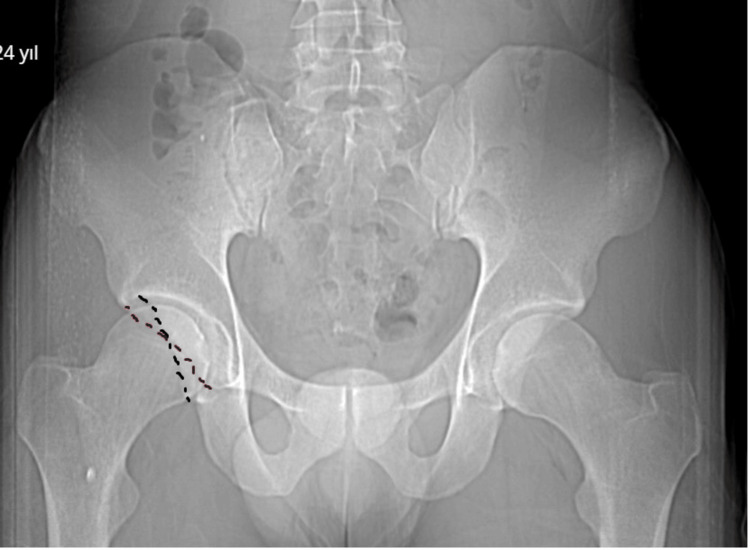
Crossover sign (CS)

Radiological evaluation of the patients was performed with an imaging system (SECTRA UniView) that allows the creation of multiplanar views. AA (Figure 1), FHNO (Figure 2), LCEA (Figure 3), and AVA (Figure 4) measurements were performed on the patients. In addition, the patients were evaluated for the presence of CS (Figure 5). AA was measured as the angle between a line drawn from the center of the femoral head to the center of the femoral neck and a line drawn to the point where the femoral head sphericity was disrupted. FHNO was measured as the distance between a line passing through the anterior cortex of the femoral neck and a line parallel to this line passing through the thickest part of the femoral head. LCEA was measured as the angle between a line drawn perpendicular to the line connecting the centers of both femoral heads and a line drawn from the center of the femoral head to the outermost rim of the acetabulum. AVA was measured as the angle between a line drawn perpendicular to the line passing through both posterior walls of the acetabulum and a line connecting the anterior and posterior walls of the acetabulum. Measurements were made on the same tomography sequences in all patients. Three months after the initial measurement, repeat measurements were performed on 30 consecutive patients to verify intra- and inter-observer reliability. Patients were evaluated in terms of measurement data, age, and sex.

Based on the measurement results, patients were classified as having a cam type if their AA was ≥ 55 or FHNO was <8; and as having a pincer type if their LCEA was ≥ 40, AVA was <15, or they had a CS. A patient was classified as having a mixed type if they met at least one cam type and at least one pincer type criteria. Patients with no out-of-range values ​​were classified as normal.

Statistical analysis

The data obtained in this study were loaded into the Statistical Product and Service Solutions (SPSS, version 22; IBM SPSS Statistics for Windows, Armonk, NY) program. In the evaluation of the data, when the parametric test assumptions were met (Kolmogrov-Smirnov), the significance test of the difference between two means was used when comparing the measurements obtained from two independent groups, when the parametric test assumptions were not met, the Man-Whitney U test was used when comparing the measurements obtained from two independent groups, and the chi-square test was used in the evaluation of the data obtained by counting in 2x2 designs and multi-pattern designs. Our data were expressed in the tables as the arithmetic mean, standard deviation, median, minimum and maximum values, number of individuals, and percentage. Values of p < 0.05 were considered to indicate statistical significance.

In this study, both intra-observer and inter-observer reliability analyses were performed. The intraclass correlation coefficients (ICC) for AA were calculated as 0.951 and 0.938; 0.910 and 0.947 for FHNO as ICC; 0.897 and 0.921 for LCEA as ICC; and 0.953 and 0.939 for AVA as ICC. In ICC measurements, values ​​less than 0.5 were taken as poor reliability, those ​​between 0.5 and 0.75 as moderate reliability, those ​​between 0.75 and 0.9 as good reliability, and those​​greater than 0.90 as excellent reliability, as suggested by Koo et al. [8]. Accordingly, all measurements were at an excellent reliability level with ICC values ​​> 0.90 in terms of both intra-observer and inter-observer reliability.

## Results

Among the 273 patients who were selected and included in the study and among the patients who underwent lower abdominal and pelvic tomography in our hospital, 160 (58.6%) were male, and 113 (41.4%) were female, with the mean age of the patients being 36.21±9.05 (20-51) years. The mean age of the male was 36.39±8.71 (20-50) years, and the mean age of the female was 35.95±9.56 (21-51) years.

CS was seen in 15% (41 patients) of the patients, but not in 85% (232 patients). This sign was seen in 11.9% (19 patients) of the men and 19.5% (22 patients) of the women. No statistically significant difference was observed between men and women in terms of the frequency of CS (p=0.089) (Table 1).

**Table 1:** Distribution of the crossover sign by sex

CS	Male	Female	Total	Conclusion
Positive, n(%)	19 (%11.9)	22 (%19.5)	41 (%15)	p=0.089
Negative, n(%)	141 (%88.1)	91 (%80.5)	232 (%85)	

In patients with CS, the mean LCEA value was 34.48°±5.89°, and the mean AVA value was 16.20°±5.79°. In patients without this sign, the mean LCEA value was 35.35°±6.71°, and the mean AVA value was 17.98°±5.04°. No statistically significant difference was found in the mean LCEA value between patients with and without this sign (p=0.43). However, a statistically significant difference was observed in the mean AVA value (p=0.04) (Table 2).

**Table 2:** Lateral centre-edge angle (LCEA) and acetabular version angle (AVA) measurements in patients with and without crossover signs (CS) *p<0.05

Paramenters	CS	N	Mean	SD	Minimum	Maximum	p value
LCEA	Positive	41	34.48	5.89	21.00	43.20	p=0.43
	Negative	232	35.35	6.71	12.60	59.80	
AVA	Positive	41	16.20	5.79	6.50	37.2	p=0.04*
	Negative	232	17.98	5.04	5.40	40.50	

As a result of the measurements, the AA average was found to be 55.43°±10.17°, the FHNO average was 8.19±1.91 mm, the LCEA average was 35.22°±6.59°, and the AVA average was 17.71°±5.19°. The AA average was 55.77°±9.95° in women and 55.19°±10.35° in men, the FHNO average was 7.94±1.84 mm in women and 8.36±1.94 mm in men, the LCEA average was 33.31°±6.71° in women and 36.57°±6.18° in men, and the AVA average was 19.75°±5.30° in women and 16.27°±4.60° in men. There was no statistically significant difference between female and male patients in terms of AA and FHNO mean values ​​(p=0.64, p=0.07, respectively). However, there was a statistically significant difference between female and male patients in terms of LCEA and AVA mean values ​​(p<0.000, p<0.00, respectively) (Table 3).

**Table 3:** Our measurement results by sex *p<0.05; AA, alpha angle; FHNO, femoral head neck offset; LCEA, lateral centre-edge angle; AVA, acetabular version angle

Parameters	Sex	N	Mean	SD	Minimum	Maximum	p value
AA	Male	160	55.19	10.35	33.70	84.00	p=0.64
	Female	113	55.77	9.95	33.20	81.10	
	Total	273	55.43	10.17	33.20	84.00	
FHNO	Male	160	8.36	1.94	5.00	14.00	p=0.07
	Female	113	7.94	1.84	5.00	12.00	
	Total	273	8.19	1.91	5.00	14.00	
LCEA	Male	160	36.57	6.18	21.00	59.8	p<0.00*
	Female	113	33.31	6.71	12.60	46.6	
	Total	273	35.22	6.59	12.60	59.80	
AVA	Male	160	16.27	4.60	5.40	29.3	p<0.00*
	Female	113	19.75	5.30	7.20	40.50	
	Total	273	17.71	5.19	5.40	40.50	

According to our measurement results, AA was measured at 55° and above in 46.5% of patients (127 patients). FHNO was measured below 8 mm in 38.8% of patients (106 patients). LCEA was measured at 40° and above in 21.6% of patients (59 patients). AVA was measured below 15° in 29.7% of patients (81 patients). AA was observed to be 55° and above in 45% of males (72 patients) and 48.6% of females (55 patients). FHNO was observed to be below 8 mm in 33.1% of males (53 patients) and 46.9% of females (53 patients). LCEA was observed to be 40° and above in 26.2% of males (42 patients) and 15% of females (17 patients). AVA was observed to be below 15° in 40% of males (64 patients) and 15% of females (17 patients). In this context, no statistically significant difference was found between males and females in terms of AA values ​​above and below the limit values ​​(p=0.5). However, a statistically significant difference was observed between male and female groups in terms of FHNA, LCEA, and AVA values ​​above and below the limit values ​​(p=0.02, p=0.02, p<0.00*, respectively) (Table 4).

**Table 4:** Measurement values by sex *p<0.05; AA, alpha angle; FHNO, femoral head neck offset; LCEA, lateral centre-edge angle; AVA, acetabular version angle

Parameters	Value	Male, n(%)	Female, n(%)	Total n(%)	p value
AA	<55°	88 (%55)	58 (%51.4)	146 (%53.5)	p=0.5
	≥55°	72 (%45)	55 (%48.6)	127 (%46.5)	
FHNO	<8 mm	53 (%33.1)	53 (%46.9)	106 (%38.8)	p=0.02*
	≥8 mm	107 (%66.9)	60 (%53.1)	167 (%61.2)	
LCEA	<40°	118 (%73.8)	96 (%85)	214 (%78.4)	p=0.02*
	≥40°	42 (%26.2)	17 (%15)	59 (%21.6)	
AVA	<15°	64 (%40)	17 (%15)	81 (%29.7)	p<0.00*
	≥15°	96 (%60)	96 (%85)	192 (%70.3)	

A strong negative correlation was found between patients with FHNO 8 mm and above and AA 55° and above (p<0.000, r=-0.749). Similarly, a negative correlation was found between patients with LCEA 40° and above and AVA 15° and above (p=0.002, r=-0.185).

In this study, there were 77 patients (28.2%) between the ages of 20-29, 89 patients (32.6%) between the ages of 30-39, and 107 patients (39.2%) between the ages of 40-51. No statistically significant difference was found between these age groups in terms of AA, AVA, and LCEA measurements (p=0.105, p=0.288, p=0.287, respectively). Statistically significant differences were observed between these age groups in terms of FHNO measurements and the presence of CS (p=0.02, p=0.009, respectively) (Table 5).

**Table 5:** Our measurement results according to age groups *p<0.05; AA, alpha angle; FHNO, femoral head neck offset; LCEA, lateral centre-edge angle; AVA, acetabular version angle

Parameters	Age	N	Mean	SD	Minimum	Maximum	p value
AA	20-29	77	56.55	10.43	39.70	84.00	p=0.105
	30-39	89	56.42	10.57	33.70	82.40	
	40-49	107	53.80	9.50	33.20	75.50	
	Total	273	55.43	10.17	33.20	84.00	
FHNO	20-29	77	7.87	2.01	5.00	14.00	p=0.02*
	30-39	89	7.98	1.79	5.00	13.00	
	40-49	107	8.58	1.87	6.00	14.00	
	Total	273	8.19	1.91	5.00	14.00	
LCEA	20-29	77	34.49	7.06	12.60	52.00	p=0.287
	30-39	89	34.94	6.02	19.40	51.10	
	40-49	107	35.97	6.68	17.60	59.80	
	Total	273	35.22	6.59	12.60	59.80	
AVA	20-29	77	17.09	5.51	5.40	37.20	p=0.288
	30-39	89	17.56	4.49	7.20	28.80	
	40-49	107	18.29	5.47	6.10	40.50	
	Total	273	17.71	5.19	5.40	40.50	

As a result of the analysis of our measurement data, the prevalence of FAI was found to be 25.3% for the cam type, 25.3% for the pincer type, and 23.8% for the mixed type. The total prevalence of FAI was found to be 74.4%.

## Discussion

There are studies reporting different results on the prevalence and characteristics of FAI in different societies and in our country. As the studies on FAI increase, awareness also increases accordingly. FAI is one of the first diagnoses that comes to mind in hip pain in young adult patients. The prevalence of this anatomical disorder in the asymptomatic population has revealed the possibility of overdiagnosis and treatment [9,10].

In studies conducted for the prevalence of FAI, measurements were made using X-ray, MRI, and CT. Studies in the literature have shown that direct radiographs may be insufficient in evaluating proximal femoral and acetabular anatomy. CT provides a clear evaluation of anatomical references and allows angle measurements to be made more reliably. In this respect, it has been stated that CT is a more reliable method compared to direct radiographs in the evaluation of FAI [11-13]. For this reason, we used CT for measurements.

Radiographic parameters used to diagnose FAI have varied among studies. There is no consensus on this issue. There are studies that generally use AA, FHNO measurements for the cam type, and AVA, LCEA, CS, and Tonnis angle measurements for the pincer type [1,2]. We used AA, FHNO, AVA, LCEA, and CS measurements because of their more comprehensive and widespread use. The cut-off values ​​of these radiographic measurements used in the diagnosis of FAI also vary among studies. For the cam type FAS, AA above 55 or 60 degrees has been accepted as significant. In addition, different values ​​have been suggested for the FHNO or FHNO ratio [4,5]. Mineta et al. stated in their study that the FHNO value may give incorrect results because the Japanese race has a small skeletal structure. Instead, they used the FHNO ratio in their study. For the pincer type, LCEA values ​​above 39 and 40 degrees were considered significant. In addition, AVA and CS were suggested for the pincer-type diagnosis [14]. In our study, we accepted AA >55°, FHNO <8 mm, LCEA >40°, and AVA <15° as significant.

When looking at the studies reporting the prevalence of FAI, it can be classified into three groups: asymptomatic and symptomatic studies and studies conducted in the general population. Some of these study examples are given in Table 6 [11,15-19]. When these studies are examined, some of them only looked at radiological parameters, and some of them only gave the prevalence of FAI. Our study is a study conducted in the general population and contributes to the literature in terms of determining the general prevalence and frequency of occurrence of morphological findings.

**Table 6:** Various studies on femoracetabular impingement (FAI) FAI, femoracetabular impingement; CT, computed tomography; MRI, magnetic resonance imaging; AA, alpha angle; FHNO, femoral head neck offset; LCEA, lateral center-edge angle; AVA, acetabular version angle; CS, crossover sign Source: Refs [11,15-19]

Studies	Number of hips	Clinic	Imaging	Measurements(mean)					FAI types (%)			FAI (%)
				AA	FHNO	LCEA	AVA	CS	cam	pincer	Mix	
Our study	273	General	CT	55.4	8.1	35.2	17.7	15	25.3	25.3	23.8	74.4
Ergen et al. [15]	68	Asymptomatic	CT	41.1	9	37.2	21.5	11.7	-	-	-	-
Mori et al. [16]	176	Symptomatic	X-ray	-	-	-	-	-	14.4	7.4	7.9	29.7
Polat et al. [11]	1076	Asymptomatic	X-ray	47.3		31	-	-	15.9	10.6	3.1	29.6
Kang et al. [17]	50	Asymptomatic	CT	45.6	9.5	34.8	18.9	40	-	-	-	-
Dickenson et al. [18]	200	General	CT	-	-	-	-	-	47	-	-	-
Reichenbach et al. [19]	244	Asymptomatic	MRI	-	-	-	-	-	24	-	-	

Frank et al. investigated the frequency of FAI imaging findings in asymptomatic hips in a systematic review of 26 studies. They found the prevalence of cam deformity to be 54.8% in athletes and 23.1% in other populations and stated that the overall cam type prevalence was 37%. They found the prevalence of pincer type to be 67% [20].

Ahn et al. evaluated 200 patients using direct radiographs in their study to determine the prevalence of FAS in asymptomatic Asian volunteers. They found the prevalence of FAI to be 38% in the cam type and 23% in the pincer type. The authors stated that the prevalence of FAI in the Asian population was similar to the prevalence of FAI in Western societies [21]. In contrast, Mosler et al. conducted direct radiographs on 445 male professional athletes to reveal ethnic differences in the prevalence of FAI. They stated that the frequency of cam deformity and acetabular dysplasia showed significant differences among ethnicities. They emphasized that this situation may arise from differences in the morphology of the femur and acetabulum in people of different ethnicities [22]. In this context, Van Houcke et al. investigated the prevalence of radiological parameters predisposing to FAI in Chinese and white subjects. They found that the prevalence of FAI was lower in the Chinese population compared to the white population [23].

When we look at our study and other studies in the literature, it is seen that there are differences in the prevalence of FAI in both the cam type and the pincer type. This supports the studies showing differences in the prevalence of FAI between societies [22,23]. Similar to this situation, when comparing other studies conducted in our country and our study, regional differences in FAI prevalence can be seen in our country [11,15]. It should not be forgotten that the fact that the studies were conducted using different imaging methods (e.g., X-ray and CT) and that the number of patients in some studies was insufficient may have been effective in the emergence of this situation.

Many of the patients who underwent FAI measurement and were found to be radiographically significant were observed to be clinically asymptomatic. This showed that the clinical-radiographic correlation was not always consistent. For this reason, the concepts of FAI and FAI syndrome (FAIS) emerged. The definition of FAIS was made in the Warwick Agreement consisting of many participants in 2016, for the definition of FAI. According to this definition, there was a consensus that patients should have appropriate symptoms, positive clinical findings, and significant imaging findings. The prognosis of the asymptomatic patient group with FAI radiological findings or whether it progresses to FAI is not clear [24]. We only investigated the radiological findings of FAI in the general population. Due to the nature of our study, we could not make a clinical distinction. This gives us the rates of occurrence of these measurements in the population. However, future studies conducted in a similar population with clinical distinction will give us an idea in terms of radiology and clinical correlation.

The high prevalence of FAI reported in studies conducted on asymptomatic populations has led to an increasing awareness of this syndrome. However, some studies have shown that this may lead to overdiagnosis [9]. In this respect, caution should be exercised in the diagnosis and treatment of FAI.

There are some deficiencies in our study. Our study is an epidemiological study, and it is generally healthier to conduct such studies in multiple centers. Our study was conducted as a single center, and this is one of the deficiencies of our study. In addition, since our study was conducted retrospectively using CT images, patients were not evaluated clinically. We believe that studies conducted with the evaluation of patients' clinical data would be more meaningful. However, our study is quite important because it is one of the most comprehensive studies conducted in our country using CT images. In addition, our study will be useful in terms of determining the pathological condition, especially in the young adult patient population presenting with hip pain complaints, and will make the surgeon's job easier.

## Conclusions

In our study, we determined the prevalence of FAI using methods with accepted diagnostic accuracy. While FAI varies among populations, it is a common morphological change in the general population. Our study found a significantly higher prevalence of FAI compared to many studies in the literature. The diagnosis of FAIS is made by clinically supporting the morphological changes observed in FAI. When making this diagnosis, the prevalence of FAI in the population can be considered to prevent unnecessary treatment.
